# Genomic and Mitochondrial Data Identify Different Species Boundaries in Aposematically Polymorphic *Eniclases* Net-Winged Beetles (Coleoptera: Lycidae)

**DOI:** 10.3390/insects10090295

**Published:** 2019-09-11

**Authors:** Matej Bocek, Michal Motyka, Dominik Kusy, Ladislav Bocak

**Affiliations:** Laboratory of Molecular Systematics, Department of Zoology, Faculty of Science, Palacky University, 17. listopadu 50, 771 46 Olomouc, Czech Republic; bocema00@gmail.com (M.B.); motyka01@gmail.com (M.M.); dominik.kusy2@gmail.com (D.K.)

**Keywords:** mtDNA, RAD, morphology, Lycidae, Metriorrhynchini, species delimitations, taxonomy

## Abstract

Species delineation is essential for any evolutionary and biodiversity research, and recent advances in genomic sequencing have made it possible to robustly define species boundaries and detect hidden diversity. Here, we studied 14 species of aposematically colored New Guinean *Eniclases* (Coleoptera: Lycidae) whose conventional morphology- and single-locus mtDNA-based taxonomy has been contentious. We analyzed mitochondrial and restriction site associated DNA fragments to obtain a phylogenetic hypothesis and compared relationships recovered by the RAD analysis with species limits based on other information. The results show the presence of cryptic diversity and common mitonuclear discordance when over 30% of individuals were incorrectly assigned to species if only mitogenomic markers were considered. Nuclear data falsified the species rank of one species and identified one earlier unrecognized lineage deserving species rank. Further, our analyses demonstrate a highly variable phenotypic differentiation, with several pairs of cryptic species standing in contrast with genetically close but phenotypically highly divergent lineages. We show that morphological and mitogenomic analyses produce reliable information for taxonomy in most cases. Nevertheless, the species boundaries among closely related species should be based on all lines of evidence, including nuclear markers.

## 1. Introduction

The delimitation of species is a basic step in alpha-diversity estimation, and accurate definitions of species limits have consequences for any subsequent research and conservation management [1,2,3,4]. The conventional morphology-based taxonomy relies on phenotypic differences and these can be affected by the convergent evolution of morphological traits, e.g., by a signaling function of some traits in the communities of unpalatable prey. Therefore, the verification of morphology-based species boundaries is highly desirable. Information on DNA structure is now easily available and the last two decades have seen intensive integration of phenotypic and molecular data in the studies dealing with large groups of closely related species [5,6,7]. Mitochondrial markers are commonly used as they usually have sufficient resolution and provide a lot of valuable information with acceptable costs of laboratory work [8,9,10]. Unfortunately, these markers do not often have sufficient resolving power for analyses of closely related species [11,12,13]. In these cases, sequencing of nuclear DNA fragments for studies on lineages in the early stage of speciation is the preferred method [14].

**Figure 1:**
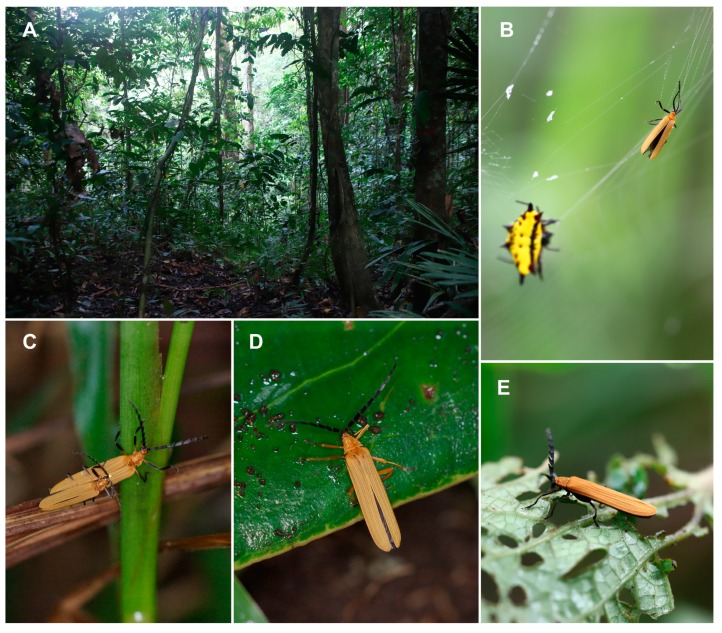
(**A**) Lowland rain forest in New Guinea; (**B**) *Eniclases* sp. in the web of *Gasteracantha* sp. in New Guinea. (**C**) *Eniclases* sp. (**D**) *Microtrichalus* sp. (**E**) *Porrostoma* sp. (Photographs © Authors).

**Figure 2:**
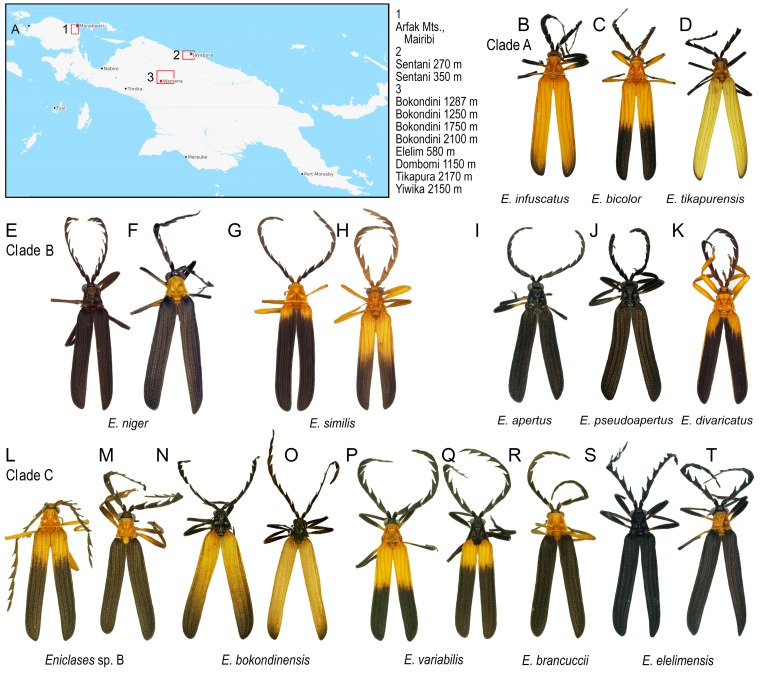
(**A**) Study area; (**B**–**T**) *Eniclases* species included in the study (Photographs © Authors).

Here, we study the metriorrhynchine genus *Eniclases* (Coleoptera: Lycidae [15]), a morphologically uniform group whose taxonomy was uncertain in some cases. Their phenotypic differences are limited to the relative size of eyes and the shape of antennae. The genitalia of most species are uniform and do not provide diagnostic characters [16]. Altogether 37 species have been described, most of them quite recently and based on the detailed morphological study [17,18,19]. A relatively extensive single-locus mtDNA dataset has recently been assembled for species from western New Guinea and the Moluccas. With molecular data, we can identify clusters of closely related species and the results indicate that morphology might not be sufficient for species delimitation [20,21]. All *Eniclases* produce pungently smelling and bitter hemolymph from ruptured membranes on antennae, legs, and elytra. They are involved in mimicry rings with other net-winged beetles, and their coloration is often variable within species (Figure 1 and Figure 2; [22]). The proper delimitation of species is crucial for discrimination between intraspecific color polymorphism and aposematic monomorphism in recently separated species.

Using the earlier produced phylogenies [22], we compare available morphological diagnostic characters, mitochondrial, and genomic data to investigate species limits in *Eniclases*. Most biodiversity research depends on morphology-based species definitions, recently often supported by a limited amount of mitochondrial DNA information. The question is how reliably species boundaries are defined using such data and if nuclear DNA data provide a congruent signal. The robust species definitions are a necessity if we build species lists, analyze their distribution, or consider the evolution of mimicry [22]. Here, we evaluate the signal from tens of thousands of nuclear loci, three mitochondrial markers, and morphology. The immerse tropical diversity cannot be always studied in such detail, so *Eniclases* serves as an example which indicates the potential pitfalls of limited information in taxonomy.

## 2. Methods

The dataset was produced for an earlier study on the structure and evolution of mimetic communities of *Eniclases* and other Metriorrhynchina in New Guinea [22]. The laboratory procedures and methods of phylogenetic analyses were described in detail there. Here, we use only data specifically related to the species delimitation. We compare earlier *cox1* mtDNA and morphology-based species boundaries [16,20] with restriction site associated DNA, i.e., RAD-based relationships and divergence [22]. The analyses of tens of thousands of nuclear loci can identify possible mitochondrial introgression and have much better resolution when closely related species and populations are considered [11,12,13]. Additionally, we include additional two additional mtDNA fragments in the analysis to verify the results obtained by an earlier *cox1*-based study [20].

### 2.1. Material and Laboratory Procedures

The material consists of 14 *Eniclases* species from localities in the Central Cordillera of Western New Guinea and Bird’s Head Peninsula (Figure 2A, Table 1, Tables S1 and S2). Specimens were fixed in 96% ethanol and total DNA was extracted using the Wizard SV96 Purification System (Promega Inc. Madison, WI, USA). The fragments *rrnL* + *tRNA-Leu* + *nad1* (~831 bp) and *nad5* + adjacent tRNAs (~1359 bp) were amplified. We used the primers that were reported by Sklenarova et al. [23] and are listed in Table S3. The PCR products were purified using PCRμ96TM Plates (MilliporeSigma Inc., Burlington, MA, USA) and sequenced by an ABI 3130 automated sequencer using the BigDye® Terminator Cycle Sequencing Kit 1.1. Sequences have been deposited in GenBank database (Accession Numbers KT265092–KT265172, MF288197–MF288482 and MG844591–MF844955). All sequences can be retrieved using voucher numbers in the format UPOL BM1234. All voucher numbers are shown in Table 1.

### 2.2. Mitochondrial DNA Data Sampling and Phylogenetic Analyses

Earlier published sequences of *cox1*, *rrnL*, and *nad5* mtDNA fragments were merged with outgroups and aligned separately using MAFFT 7.017 [24,25] in Geneious 7.1.9 (http://www.geneious.com). We used IQ-TREE 1.6.11 [26] to estimate mtDNA phylogeny with the ultrafast bootstrap support (UFboot) set to 5000 iterations. The best models for mtDNA fragments were selected using ModelFinder [27] implemented in IQ-TREE (Table S4). Additionally, uncorrected pairwise distances were counted in Geneious 7.1.9.

### 2.3. Next-RAD Sampling, Filtering and SNP Calling

In total, 66 individuals were included in subsequent restriction-digest-associated DNA sequencing (RADseq). The samples represent all available species of *Eniclases* from 7 localities in New Guinea. We used de novo assembly with a specific clustering threshold (Wclust, degree of sequence similarity) to search for orthologous sequences [28]. The whole genome RAD genomic sequencing was provided by SNPsaurus Inc. and the Illumina Hi-Seq system was used to generate data. Illumina reads have been deposited in Sequence Read Archive (SRA; http://www.ncbi.nlm.nih.gov/sra; SRA data ABC123456).

Using iPYRAD v. 0.6.24 [29,30] we demultiplexed, trimmed, and filtered RAD reads and de novo assembled orthologous loci. The maximum size of the data matrix varied as we increased or decreased the Wclust parameter. We tested the matrices by analyzing five Wclust values from 0.7 by increasing 0.05 for each filtering. To keep the high proportion of potential loci accepted and the possible highest rate of sample heterozygosity, the Wclust of 0.85 was used for the analysis presented here. A minimum depth (MinDepth) of six reads was used together with a minimum number of four samples that contain data in a given locus in the final dataset (MinCov). Whereas the proportion of missing data and number of loci filtered are strongly dependent on the parameter of MinCov, we also produced other data matrices with specific MinCov (8, 16, 33, 48 and 60,) and Wclust (from 0.7 to 0.9). Additionally, data were filtered independently for clades A–C as defined by whole-data analyses. These analyses were conducted to compare intra-clade topologies recovered by the analyses of differently assembled datasets.

### 2.4. RAD Tree Processing

Each of a single matrix generated with a specific Wclust and MinCov value discussed earlier was used to infer a phylogenetic tree. The matrices were analyzed by maximum likelihood approach using IQ-TREE with 5000 ultrafast bootstrap (UFboot) iterations. ModelFinder implemented in IQ-TREE estimated optimal model of evolution for each matrix. The resultant tree topologies from all data matrices were subsequently examined.

### 2.5. Morphology

Morphological characters were observed for all sequenced specimens. The body length, pronotum width and length, maximum diameter of eyes in the lateral view (EDiam), and the minimum interocular distance in the frontal part of cranium (EDist) were taken using an ocular scale. Color patterns of the pronotum and elytra, shape of pronotum and antennae, and structure of the elytral costae were documented by photographs which were taken using a binocular microscope Olympus SZX-16 and a Canon EOS digital camera. The individual shots were assembled in Helicon Focus 6 (www.heliconsoft.com). Due to the previously reported uniformity of the genitalia of both sexes [16], only some individuals were dissected, but the illustrations were not produced.

## 3. Results

### 3.1. Morphology

Most *Eniclases* are phenotypically similar (Figure 2B–T). The color patterns are simple and the upper side of the body can be bicolored, uniform black, or yellow. Two colors, bright and dark, are present in the pronotum and elytra of most species. The bright colors include pale yellow and bright yellow to orange; the dark parts are black to blackish brown. The transition between bright and dark parts of elytra can be gradual or clearly defined. The body size varies between 6.3 and 11.6 mm, the male eyes are relatively small, sometimes the eye distance is larger than the eye diameter. The uniformly black colored species have usually large eyes; their eye diameter is up to 1.4 times the interocular distance (Table 2). The antennae are weakly serrate to flabellate in extremes, but most species have moderately serrate antennae. Male genitalia are uniform in all species. The phallus is slender, mostly membranous in apical two thirds, only the dorsal line and the tip of the phallus are weakly sclerotized and pigmented. Parameres are absent, and the phallobase is circular with a translucent membrane. The internal sac is completely membranous, without any thorns or large setae.

### 3.2. MtDNA Analysis

**Figure 3:**
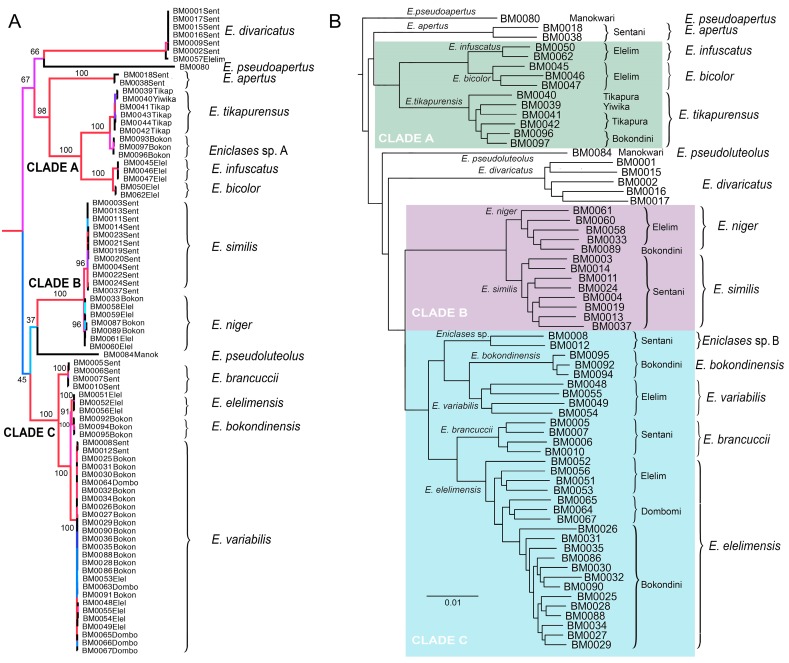
Maximum likelihood phylogenies of *Eniclases*. Nodes are colored according to ultrafast bootstrap values. The clades A, B and C designate lineages of closely related species whose delimitation is discussed in the text. (**A**) topology recovered by the analysis of three mitochondrial DNA fragments; (**B**) topology recovered by the analysis of RAD dataset (Wclust = 0.85, MinCov = 4). Species limits follow Bocak and Bocek (2016) in Figure (**A**) and the results of RAD analysis in Figure (**B**). Labels at terminals designate voucher numbers and geographic origin.

Maximum likelihood mitochondrial analyses support a division into three separate deeply rooted species and three clades of closely related species: (1) clade A (BS 100%)—*E. infuscatus*, *E. bicolor*, *Eniclases* sp. A, and *E. tikapurensis*; (2) clade B (BS 100%)—*E. niger* and *E. similis*, and (3) clade C (BS 100%)—*E. brancuccii*, *E. elelimensis*, *E. bokondinensis* and *E. variabilis*. The clades represent closely related species with mtDNA interspecific uncorrected pairwise divergence of 0.45%–6.45%. The *cox1* mtDNA uncorrected pairwise distances are shown in Table 3). All constituting species occur in the Central Highlands and adjacent lowlands at the northern coast of New Guinea (Figure 2A, groups of localities 2 and 3). The deeply rooted species are represented by *E. pseudoluteolus* from mountain forests in the Central Highlands, *E. divaricatus*, a widely distributed species from northern coastal lowlands, and *E. pseudoapertus* from the Bird’s Head Peninsula. Deeper splits in the tree are poorly supported and as they do not have any relevance for species delineation, they are not discussed further (Figure 3A).

### 3.3. Next-RAD Analysis and Phylogeny

The nuclear RAD dataset encompasses 86,692 possible loci, 649.9 thousand SNPs sequenced with a minimum depth 6. We used Wclust = 0.85, which represents a compromise between the number of RAD clusters obtained and the individual heterozygosity. Various values of Wclust and MinCov produced dataset with variable number of loci and data completeness and the topology remained stable (results were shown in reference [22]).

**Figure 4:**
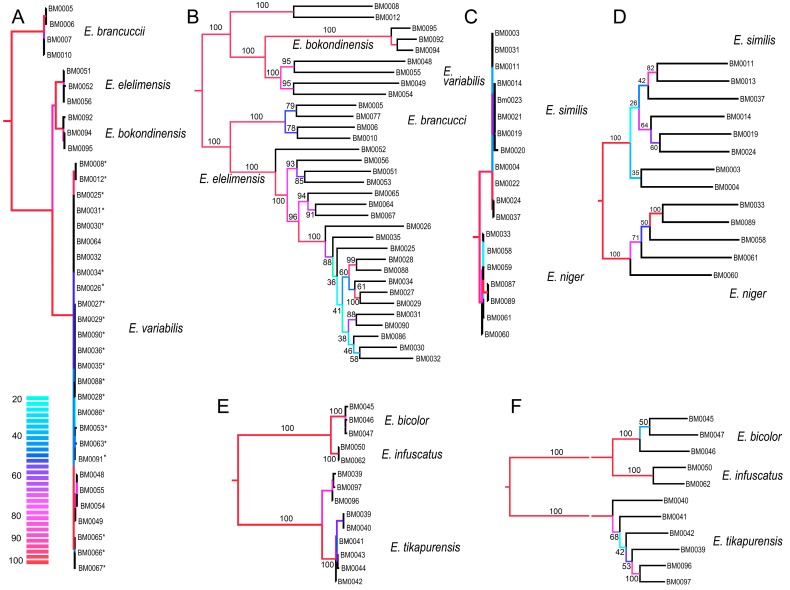
(**A**,**C**,**E**) Phylogenetic relationships recovered by the analysis of the concatenated mitochondrial DNA dataset; (**B**,**D**,**F**) The phylogenetic relationships recovered by the analysis of the RAD dataset when data for each clade are separately filtered and analyzed (Wclust = 0.85, MinCov = 4). Values at branches designate ultrafast bootstrap values. (**C**) The dated phylogenetic tree based on mutation rates of three mitochondrial fragments; (**D**,**E**) The geographic position of sampled localities. The color scale designates ultrafast bootstrap values in mtDNA topologies.

**Figure 5:**
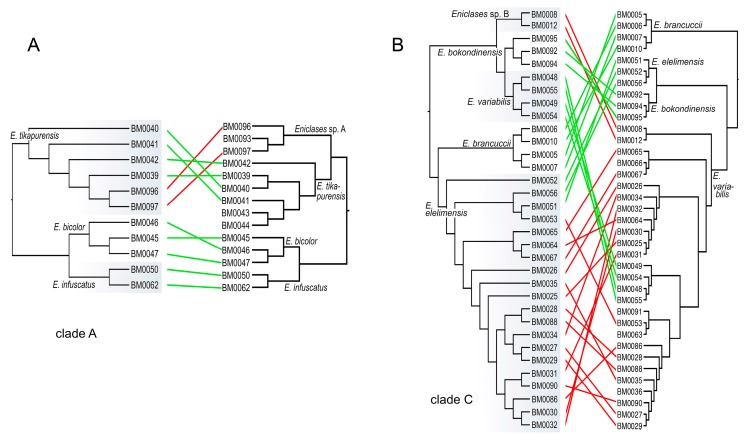
The summary of the identification of individuals in clades A and C using RAD (**left trees**) and mitochondrial (**right trees**) phylogenetic analyses. The red links designate specimens assigned to different species, green links the individuals assigned to the same species by both analyses.

The presented RAD analysis yields a highly supported phylogenetic tree that slightly differs from those recovered by the mitochondrial tree (Figure 3, Figure 4 and Figure 5). *Eniclases sp. A* forms a terminal branch in the *E. tikapurensis* clade (Figure 3B). The clade of BM0008 and BM0012 is identified as an independent deep branch; this clade is designated as species *Eniclases sp. B* hereafter. The RAD-based composition of the species-rank clades *E. variabilis* and *E. elelimensis* differs substantially from those defined based on mtDNA data (Figure 3A,B and (Figure 5). The genomic data suggest a restricted concept of *E. variabilis* and a high number of individuals is recovered within the *E. elelimensis* clade. 

## 4. Discussion

The conventional alpha-taxonomy of *Eniclases* has been based on morphology and always depended on a limited number of diagnostic traits [16,17,18,31]. The genitalia of *Eniclases* are very uniform and cannot be used for reliable identification. Therefore, most species were defined based on differences in the relative size of male eyes, the shape of male antennae, and body coloration, which is a highly questionable characteristic as shown in Figure 2B–T. Although additional differences can be found in the structure of pronotal carinae and elytral costae, these are subtle and intra-specifically highly variable in *Eniclases* (Figure 2) [16]. Additionally, the phenotypic characters might be differently selected, i.e., the local populations might slightly differ morphologically due to putative interaction with different local co-mimics [22]. Due to the high level of uncertainty and a limited number of available individuals, the species were conservatively defined in the earlier morphological study and the authors noted the presence of putative cryptic diversity [16]. Despite recent intensive field research, we still do not have enough material to analyze the morphology of *Eniclases* in detail and we have to limit morphological information to the relative size of male eyes, the shape of antennae, the body size.

*Eniclases* occur in localities with a high number of net-winged beetle species. As all net-winged beetles are unpalatable and aposematically colored, it is believed that the observed phenotypic similarity of unrelated species is caused by the selection for monomorphism (Figure 2B–T) [22,32,33,34,35,36]. The phenotypic resemblance is close even between species from different genera (Figure 1C,D) and it poses a serious problem if closely related species of *Eniclases* are compared. Recently, some *Eniclases* were sequenced for a *cox1* mtDNA fragment, while new species were described with a species rank given to sets of phenotypically distinguishable populations without intermediates or sets of individuals identified as robust genetic clusters in the mitochondrial phylogeny [20]. The Bayesian Poisson Tree Process was used by the authors along with a genetic distance method and morphology. The reasons for the delimitation of each species were justified in detail [20]. The main illustration with posterior probabilities for critical bipartitions is reprinted from Bocek and Bocak [20] in Figure S5.

Here, we use multi-locus RAD data to verify the earlier proposed species limits. We apply the genotypic cluster definition [37]. The independent characters which distinguish these clusters need to be correlated with each other. We request the concordance between two independent data sources for the definition of two sister species; here they are the morphological diagnostic traits and genomic loci. If species are distantly related, e.g., non-sister relationships if they belong to distant clades, then we accept as indicative the concordance between mitochondrial and genomic data and accept high phenotypic similarity (e.g., *E. bicolor* and *E. similis* or *Eniclases sp. B* and *E. elelimensis*; Figure 3 and Figure 4). We identify profound mitonuclear discordance in several closely related species (always within a single clade with maximum *cox1* mtDNA divergence < 4.0%; Table 2, Figure 3, Figure 4 and Figure 5), possibly caused by mitochondrial introgression or incipient sorting of mitochondrial haplotypes. The mitogenomic incongruence suggests interspecific mating, but not necessarily compromised genetic cluster coherence [12]. As a result, separate analyses of mitochondrial and nuclear loci indicate different species limits (Figure 3 and Figure 4). We prefer the results of the RAD analysis (Figure 3B and Figure 4B,D,E) as the nuclear genome-wide data can better resolve the clusters of individuals with shared ancestry, especially among the closely related species and recently separated populations [12,13,38,39,40]. The robustness of RAD analyses was tested by the filtering and analysis of all data and additional separate filtering and *de novo* assembling of datasets for clades A, B, and C (Figure 3 and Figure 4B,D,F). Additionally, the robustness of the presented topology is supported by the congruence between topology and geographic origins (Figure 3B). The whole-data analyses using various combinations of Wclust and MinCov values were reported by Bocek et al. [22].

### 4.1. Species Delimitation: Congruence and Conflicts

We analyzed 14 species altogether (13 of them formally named) and based on evidence from RAD phylogenetics, we identified the false assignment of species rank or different species limits for four of them (Table 1, Figure 3, Figure 4 and Figure 5). In total, over 30% of individuals were assigned to a different species using mitogenomic and nuclear genomic data. Regardless of the identified differences, the present RAD analyses do not result in any formal taxonomic changes and all formally described species remain valid [16,20].

The incongruent signal from uniform morphology and divergent mtDNA was identified in *E. tikapurensis* (Figure 3, Figure 4 and Figure 5, Table 3; *cox1* mtDNA divergence 1.18%). The mtDNA analysis suggests two reciprocally monophyletic lineages designated as *E. tikapurensis* from Tikapura and Yiwika and *Eniclases sp. A* from Bokondini ([20]; the minimum geographic distance 10.7 km; Table S2). In the genomic analysis, the Bokondini population has a terminal position concerning the populations from Yiwika and Tikapura (Figure 3A,B and Figure 4E,F). We prefer the results of RAD analyses and assign all populations as a single species, *E. tikapurensis*. As these populations are morphologically uniform, the Bokondini population was only informally designated as *Eniclases sp. A* [20]. Therefore, no taxonomic change is needed [20].

Two individuals, BM008 and BM0012 from Sentani in the Cyclop Mts., share mitochondrial genome with *E. variabilis* from Elelim and *E. elelimensis* from Elelim, Bokondini, and Dombomi. Using whole-genome RAD data, these two individuals were recovered as a separate lineage in a sister position with other species of the clade C (Figure 3, Figure 4A,B, and Figure 5B) and it should obtain species rank as an additional cryptic lineage. We prefer to postpone the formal description as it can only be identified with nuclear genomic data, and a comprehensive study with additional samples should definitively solve its limits and relationships with *Eniclases* from additional localities (compare mtDNA and RAD-based phylogenies in Figure 4A,B).

As noted above, some *E. variabilis* (Figure 2P,Q) and *E. elelimensis* (Figure 2S,T) share a highly similar mtDNA. Nuclear genomic data are needed to separate them if their identification should not exclusively rely on coloration, which is a questionable character in other cases (Figure 2 and Figure 3). Both species are color polymorphic, but *E. variabilis* contains only individuals with a brightly colored humeral part of elytra and the redefined *E. elelimensis* individuals with completely black elytra (Figure 2P,Q,S,T). The RAD analysis recovered similarly colored *E. brancuccii* as a sister species to *E. elelimensis* (Figure 1B). Hence, only the results obtained from RAD analyses are congruent with the distribution of aposematic patterns.

The genetically distinct *E. bicolor* and *E. infuscatus* occur sympatrically in Elelim and besides the color patterns, they only slightly differ in the relative size of eyes (Figure 4A,D; Table 2; [20]). Their low-level mtDNA distinctness (uncorrected pairwise divergence 1.09%) is now robustly supported by genomic data.

Two species pairs representing the cryptic species *E. bicolor* cannot be morphologically separated from sympatric but distantly related *E. similis.* These species belong to the clades A and B, respectively, and differ only in mtDNA and genomic markers (Figure 3 and Figure 4E,F). A further species pair, *E. brancuccii* and allopatrically distributed *E. elelimensis,* both belonging to the clade C, are morphologically indistinguishable, but genetically divergent at the similar level as some sister species pairs which display a phenotypical differentiation (Figure 1B; Table 2). The nuclear data suggest their sister relationships in conflict with mitogenomic data which indicate distant positions (Figure 3, Figure 4 and Figure 5). In this case, the data obtained are difficult to interpret and we have to base our delimitation on potentially incomplete sampling. An additional survey across the whole range might question the integrity of these species, as high geographically structured divergence has been documented in net-winged beetles [9].

Additionally, we assign the species status to allopatric *E. niger* and *E. similis*. Their genetic *cox1* mtDNA divergence is very low (0.45%, Table 3) and the single-locus analyses suggested paraphyly of *E. niger* ([20]; Figures S2–S4). Only the present three-fragment mtDNA analysis weakly supports their reciprocate monophyly (Figure 3A and Figure 4C). Genomic data affirm both the distinctness and monophyly, although at a very low level of divergence (Figure 3, Figure 4 and Figure 5). The possibility that these two species represent geographic isolates whose distinctness is caused by incomplete sampling is falsified by their divergence in the relative size of eyes (Table 2). Additionally, they differ in color patterns (Figure 1E–H). The differences in the relative size of eyes and coloration may indicate different diel (day-night) activity, i.e., species’ separation of activity times. The dark-colored *Eniclases* have regularly large eyes (Table 2), which are characteristic for beetles with night activity [41].

### 4.2. Taxonomy of Eniclases

Only seven species of *Eniclases* were described before a large collection of Papuan net-winged beetle fauna was studied in the early 1990s [16,17,18,31]. Earlier studies relied on external characters, only a limited number of species was recognized, and, probably, cryptic species were not detected. The first molecular data were recently used for *Eniclases* identification and the number of nominal species was recently raised to thirty-seven [20,21]. The conservative taxonomic approach was applied when only one mtDNA fragment was available and species rank for allopatric taxa was given only when the genetic diversification was accompanied by phenotypic differences. The present analysis of nuclear loci identified substantial inconsistencies in mtDNA and nDNA-based species limits (Figure 5). Almost a third of individuals is assigned to a different species with nuclear loci (21 of 66 individuals) and the incorrectly identified individuals are concentrated in clades of closely related species (Figure 5). No formal taxonomic changes have to be proposed in *Eniclases* despite such mitonuclear incongruence. Simultaneously, we show that the delimitation of species using only on mitochondrial DNA without any support from phenetic divergence is not advisable.

Here, we simultaneously consider all DNA data to separate morphologically similar species that keep genetic distinctness in sympatry and to assign the status of either a species or geographic isolate to allopatrically distributed populations. The distinct genetic clusters occurring sympatrically in a single locality should obtain species rank under most species concepts (indirect evidence for the absence of interspecific breeding [37,42,43,44]). Nevertheless, the continuous character of diversification makes the decision which populations should be given the rank of a species, subspecies or divergent population sometimes disputable, especially in the case of allopatric populations and incomplete sampling [43,45,46,47,48].

We tried to simultaneously evaluate mitochondrial and genomic genetic divergence and differences in several identified diagnostic characters to define species as natural evolutionary independent units and to avoid the delimitation of species, which would represent artificial assemblages of unrelated phenotypically similar populations. Although such an approach cannot be designated as an integrative taxonomy due to the absence of rigorous morphological analysis, it is the best possible approach when a limited number of samples is available and when *Eniclases* can be in different places under various selective pressures due to variable structure of mimetic communities [22]. Unfortunately, our combined approach does not produce an easily manageable taxonomic system. Under the present state of knowledge, the robust assignment of some populations to a species needs the simultaneous examination of the morphology and nuclear DNA. *Eniclases* seems to be a taxonomist’s nightmare and the problems with practical identification are not limited to sister taxa, but include also distantly related species. Our results confirm the complexity of the speciation process and that some level of arbitrariness is unavoidable even if extensive data is available for populations in an early phase of diversification [44,46,47,49].

## 5. Conclusions

The combination of morphological approach, mtDNA and RAD phylogenies provides evidence for unlinked genetic and phenotypic differentiation and the complex speciation with incomplete lineage sorting or high levels of introgression in the early phase of lineage divergence. Diagnostic phenotypic characters are undoubtedly important for end-users of the formal classification and we wholeheartedly prefer pragmatically defined and easily diagnosable species. Nevertheless, our detailed study whole-genome data shows that some species can only be diagnosed using all data, including genomic markers. We identified cryptic species, both closely and distantly related, high genetic divergence between geographically close populations, and genetically close sister species with apparent phenotypic divergence. Although sometimes insufficient, the combination of morphology and mitochondrion is an economically feasible approximation, which can reliably identify most species. The species limits should be verified when the species are closely related and phenotypic divergence is very low. Then, multi-locus genomic data grant an efficient way to study taxonomy and rigorously define species boundaries.

The molecular studies identified unsuspected amounts of genetic diversity among *Eniclases* in the limited area of New Guinean Central Highlands. Most species were collected in a single locality and although our sampling is surely incomplete, high turnover is characteristic for these beetles. The results obtained indicate that only a relatively dense network of protected areas can preserve the genetic and species diversity of such groups.

## Figures and Tables

**Table 1:** The list of species, their geographic origins, collecting circumstances and aposematic patterns localities. Individuals with incongruent species placement in mtDNA and restriction site associated DNA analyses; P Individuals belonging to a polymorphic species based on the restriction site associated DNA phylogeny.

*Eniclases* Identification Genomic		mtDNA and morph.	Voucher Number (UPOL+)	Locality (All New Guinea; See Figure 2A)
*E. pseudoapertus*		*E. pseudoapertus*	BM0080	Arfak Mts., Maibri, 1570 m
*E. pseudoluteolus*		*E. pseudoluteolus*	BM0084	Arfak Mts., Maibri, 1570 m
*E. divaricatus*		*E. divaricatus*	BM0057	Elelim, 580 m
*E. divaricatus*		*E. divaricatus*	BM0001, 02, 09, 15–17	Sentani, 275 m
*E. apertus*		*E. apertus*	BM0038	Sentani, 360 m
*E. apertus*		*E. apertus*	BM0018	Sentani, 275 m
*E. niger*	P	*E. niger*	BM0087, 89	Bokondini, 1287 m
*E. niger*	P	*E. niger*	BM0058–61	Elelim, 580 m
*E. similis*	P	*E. similis*	BM0024	Sentani, 275 m
*E. similis*	P	*E. similis*	BM0037	Sentani, 360 m
*E. similis*	P	*E. similis*	BM0003–04, 19–23, 11, 13–14	Sentani, 275 m
*E. infuscatus*		*E. infuscatus*	BM0050	Elelim, 580 m
*E. infuscatus*		*E. infuscatus*	BM0062	Elelim, 650 m
*E. bicolor*		*E. bicolor*	BM0045–47	Elelim, 580 m
*E. tikapurensis*		*Eniclases* sp. A	BM0093	Bokondini, 1750–1900 m
*E. tikapurensis*		*Eniclases* sp. A	BM0096–97	Bokondini, 2100 m
*E. tikapurensis*		*E. tikapurensis*	BM0039	Yiwika, 2100 m
*E. tikapurensis*		*E. tikapurensis*	BM0040–44	Tikapura, 2170 m
*Eniclases* sp. B	P	*E. variabilis*	BM0008, 12	Sentani, 275 m
*E. brancuccii*		*E. brancuccii*	BM0005–07, 10	Sentani, 275 m
*E. bokondinensis*		*E. bokondinensis*	BM0092, 94–95	Bokondini, 1750–1900 m
*E. variabilis*	P	*E. variabilis*	BM0048–49, 54–55	Elelim, 580 m
*E. elelimensis*	P	*E. elelimensis*	BM0051–52, 56	Elelim, 580 m
*E. elelimensis*	P	*E. variabilis*	BM0053	Elelim, 580 m
*E. elelimensis*	P	*E. variabilis*	BM0027–29, 35–36	Bokondini, 1250–1300 m
*E. elelimensis*	P	*E. variabilis*	BM0086, 88, 90–91	Bokondini, 1287 m
*E. elelimensis*	P	*E. variabilis*	BM0063–67	Dombomi, 1150 m
*E. elelimensis*	P	*E. variabilis*	BM0025–26, 3–32, 34	Bokondini1250–1300 m

**Table 2:** Measurements of *Eniclases* included in the current DNA analyses. (n.a.—not available. Abbreviation: EDiam—diameter of eyes, EDist—distance between eyes.).

Species	Body Length	Width Humeri	Pronotum Length	Width	EDiam/EDist Male
*E. pseudoapertus*	6.3	1.6	0.75	1.2	1.4
*E. divaricatus*	6.8–9.7	2.1–2.3	1.2–1.3	1.7–1.7	0.92–0.96
*E. pseudoluteolus*	9.3	2.3	1.15	1.6	0.9
*E. apertus*	5.7–8.4	1.34–1.7	0.9	1.25	1.15–1.17
*E. tikapurensis*	9.5–11.1	2.0–2.5	1.1–1.3	1.4–1.7	1.11–1.40
*E. bicolor*	10.3	2.4	1.4	1.7	n.a.
*E. infuscatus*	12.1	2.5	1.25	1.6	n.a.
*E. brancuccii*	7.6–8.0	1.8–1.9	1.0–1.1	1.5–1.8	1.0
*E. bokondinensis*	9.2	2.05	1.0	1.35	n.a.
*E. elelimensis*	6.9–8.1	1.5–1.9	0.9–1.1	1.3–1.4	n.a.
*E. variabilis*	6.6–8.2	1.6–2.0	0.1–1.1	1.1–1.35	0.83–0.95
*E. niger*	9.2–11.6	2.2–2.8	1.3–1.6	1.3–1.6	1.17–1.28
*E. similis*	7.5–9.7	1.9–2.3	1.1–1.4	1.8	1.02–1.1

**Table 3:** The *cox1* mtDNA uncorrected pairwise distances among species-rank lineages. The designation after slash refers to the mtDNA and morphology defined species. The highlighted values designate genetic distances within clades of closely related species and population (see Figure 3 and text for the reference on the clades A–C). Abbreviations in the top line are derived from the species names in the second column. If two species designations are given, they refer to mtDNA and RAD-based species limits, respectively (See Figure 3A,B).

	div	sim	nig	bra	var/B	var	elel	bok	ape	tik	tik/A	bic	inf	psl	psap
BM0001 *E. divaricatus*	-														
BM0003 *E. similis*	12.08														
BM0033 *E. niger*	12.35	0.45	-												
BM0005 *E. brancuccii*	12.53	8.63	8.17	-											
BM0008 *E. sp. B/variabilis*	12.72	8.63	8.17	3.91	-										
BM0054 *E. variabilis*	12.72	8.54	8.08	4.27	0.45	-									
BM0051 *E. elelimensis*	12.72	8.08	7.63	3.81	1.54	1.63	-								
BM0092 *E. bokondinensis*	13.08	8.17	7.72	4	1.73	1.82	0.73	-							
BM0018 *E. apertus*	13.17	10.26	10.17	10.08	10.35	10.54	9.72	10.08	-						
BM0039 *E. tikapurensis*	13.17	10.08	9.99	9.81	9.99	10.17	8.99	9.45	6.81	-					
BM0093 *E. tikapur./sp.A*	13.62	9.81	9.72	9.9	9.9	9.99	9.08	9.54	7.27	1.18	-				
BM0045 *E. bicolor*	12.90	10.54	10.45	9.63	9.81	9.9	8.99	9.08	8.72	6.45	6.27	-			
BM0050 *E. infuscatus*	12.72	9.99	10.08	9.45	9.54	9.54	8.54	8.63	8.72	6.18	6.18	1.09	-		
BM0084 *E. pseudoluteolus*	12.99	9.45	9.26	10.26	10.9	10.81	9.81	10.26	12.08	10.81	11.17	11.81	11.26	-	
BM0080 *E. pseudoapertus*	13.26	10.9	10.9	10.9	11.44	11.35	10.99	11.44	11.44	11.35	11.35	11.08	10.63	11.53	-

## Supplementary Materials

The following are available online at https://www.mdpi.com/2075-4450/10/9/295/s1. Supplementary Text. The brief taxonomic history, morphology, and diversity of *Eniclases* Waterhouse, 1879. Table S1. The list of sampled localities with coordinates. Table S2. Euclidian distances among sampled localities in central New Guinea. Table S3. The list of primers used for mtDNA amplification. Table S4. Characteristics of datasets and best-fit models for mtDNA and nextRAD partitions. Figure S1. Topology recovered by the analysis of three mitochondrial DNA fragments, outgroups shown. Figure S2. The phylogenetic tree recovered by the analysis of *cox1* mtDNA fragment. Figure S3. The phylogenetic tree recovered by the analysis of *rrnL* mtDNA fragment. Figure S4. The phylogenetic tree recovered by the analysis of nad5 mtDNA fragment. Figure S5. Bayesian Poisson Tree Process species delimitation using *cox1* mtDNA data (reprinted from Bocek & Bocak 2016).

## References

[1] DeSalle R., Egan M.G., Siddall M. (2005). The unholy trinity: Taxonomy, species delimitation and DNA barcoding. Phil. Trans. R. Soc. Biol. Sci..

[2] Larson W.A., Seeb L.W., Everett M.V., Waples R.K., Templin W.D., Seeb J.E. (2014). Genotyping by sequencing resolves shallow population structure to inform conservation of Chinook salmon (Oncorhynchus tshawytscha). Evol. Appl..

[3] Nater A., Mattle-Greminger M.P., Nurcahyo A., Nowak M.G., de Manuel M., Desai T., Groves C., Pybus M., Sonay T.B., Roos C. (2017). Morphometric, behavioral, and genomic evidence for a new orangutan species. Curr. Biol..

[4] Abdelkrim J., Aznar-Cormano L., Buge B., Fedosov A., Kantor Y., Zaharias P., Puillandre N. (2018). Delimiting species of marine gastropods (Turridae, Conoidea) using RAD sequencing in an integrative taxonomy framework. Mol. Ecol..

[5] Riedel A., Sagata K., Surbakti S., Tanzler R., Balke M. (2013). One hundred and one new species of *Trigonopterus* weevils from New Guinea. ZooKeys.

[6] Ahrens D., Monaghan M.T., Vogler A.P. (2007). DNA-based taxonomy for associating adults and larvae in multi-species assemblages of chafers (Coleoptera: Scarabaeidae). Mol. Phyl. Evol..

[7] Ahrens D., Fujisawa T., Krammer H.-J., Eberle J., Fabrizi S., Vogler A.P. (2016). Rarity and incomplete sampling in DNA-based species delimitation. Syst. Biol..

[8] Riedel A., Tanzler R., Pons J., Suhardjono Y.R., Balke M. (2016). Large-scale molecular phylogeny of Cryptorhynchinae (Coleoptera, Curculionidae) from multiple genes suggests American origin and later Australian radiation. Syst. Entomol..

[9] Li Y., Gunter N., Hong P., Bocak L. (2015). DNA-based species delimitation separates highly divergent populations within morphologically coherent clades of poorly dispersing beetles. Zool. J. Linn. Soc..

[10] Morinière J., Cancian de Araujo B., Lam A.W., Hausmann A., Balke M., Schmidt S., Hendrich L., Doczkal D., Fartmann B., Arvidsson S. (2016). Species identification in malaise trap samples by DNA barcoding based on NGS technologies and a scoring matrix. PLoS ONE.

[11] Cruaud A., Gautier M., Galan M., Foucaud J., Saune L., Genson G., Dubois E., Nidelet S., Deuve T., Rasplus J.-Y. (2014). Empirical assessment of RAD sequencing for interspecific phylogeny. Mol. Biol. Evol..

[12] Bray T.C., Bocak L. (2016). Slowly dispersing neotenic beetles can speciate on a penny coin and generate space-limited diversity in the tropical mountains. Sci. Rep..

[13] Kobayashi T., Sota T. (2019). Divergent host use among cryptic species in the fungivorous ciid beetle Octotemnus laminifrons (Motschulsky, 1860), with descriptions of three new species from Japan. Syst. Entomol..

[14] Herrera S., Shank T.M. (2016). RAD sequencing enables unprecedented phylogenetic resolution and objective species delimitation in recalcitrant divergent taxa. Mol. Phyl. Evol..

[15] Sklenarova K., Kubecek V., Bocak L. (2014). Subtribal classification of Metriorrhynchini (Insecta: Coleoptera: Lycidae): An integrative approach using molecular phylogeny and morphology of adults and larvae. Arthr. Syst. Phyl..

[16] Bocak L., Bocakova M. (1991). Revision of the genus Eniclases Waterhouse, 1879 (Coleoptera, Lycidae, Metriorrhynchinae). Mitt. Münch. Entomol. Ges..

[17] Waterhouse C.O. (1879). Illustration of the Typical Specimens of Coleoptera in the Collection of the British Museum. Part I.—Lycidae.

[18] Pic M. (1921). Contribution à l’étude des Lycides. L’Echange.

[19] Kleine R. (1926). Coleoptera—Lycidae. Nova Guin..

[20] Bocek M., Bocak L. (2016). Species limits in polymorphic mimetic Eniclases net-winged beetles from New Guinean mountains (Coleoptera: Lycidae). Zookeys.

[21] Bocek M., Adamkova K. (2019). New species of trichaline net-winged beetles, with remarks on the phylogenetic position and distribution of Schizotrichalus (Coleoptera: Lycidae: Metriorrhynchinae). Zootaxa.

[22] Bocek M., Kusy D., Motyka M., Bocak L. Persistence of multiple patterns and intraspecific polymorphism in multi-species Müllerian communities of net-winged beetles. Front. Zool..

[23] Sklenarova K., Chesters D., Bocak L. (2013). Phylogeography of poorly dispersing net-winged beetles: A role of drifting India in the origin of Afrotropical and Oriental fauna. PLoS ONE.

[24] Katoh K., Standley D.M. (2013). MAFFT Multiple Sequence Alignment Software Version 7: Improvements in performance and usability. Mol. Biol. Evol..

[25] Katoh K., Toh H. (2008). Improved accuracy of multiple ncRNA alignment by incorporating structural information into a MAFFT-based framework. BMC Bioinform..

[26] Nguyen L.T., Schmidt H.A., Von Haeseler A., Minh B.Q. (2015). IQ-TREE: A fast and effective stochastic algorithm for estimating maximum-likelihood phylogenies. Mol. Biol. Evol..

[27] Kalyaanamoorthy S., Minh B.Q., Wong T.K.F., Von Haeseler A., Jermiin L.S. (2017). ModelFinder: Fast model selection for accurate phylogenetic estimates. Nat. Meth..

[28] Ekblom R., Galindo J. (2011). Applications of next generation sequencing in molecular ecology of non-model organisms. Heredity.

[29] Eaton D.A.R. (2014). PyRAD: assembly of de novo RADseq loci for phylogenetic analyses. Bioinformatics.

[30] Eaton D.A.R., Overcast I. (2016). iPYRAD: Interactive Assembly and Analysis of RADseq Data Sets. https://ipyrad.readthedocs.io/.

[31] Linsley E.G., Eisner T., Klots A.B. (1961). Mimetic assemblages of sibling species of lycid beetles. Evolution.

[32] Eisner T., Kafatos F.C., Linsley E.G. (1962). Lycid predation by mimetic adult Cerambycidae (Coleoptera). Evolution.

[33] Moore B.P., Brown W.V. (1981). Identification of warning odour components, bitter principles and antifeedants in an aposematic beetle: Metriorrhynchus rhipidius (Coleoptera: Lycidae). Ins. Biochem..

[34] Eisner T., Schroeder F.C., Snyder N., Grant J.B., Aneshansley D.J., Utterback D., Meinwald J., Eisner M. (2008). Defensive chemistry of lycid beetles and of mimetic cerambycid beetles that feed on them. Chemoecology.

[35] Bocak L., Yagi T. (2010). Evolution of mimicry patterns in Metriorrhynchus (Coleoptera, Lycidae): E history of dispersal and speciation in Southeast Asia. Evolution.

[36] Motyka M., Kampova L., Bocak L. (2018). Phylogeny and evolution of Müllerian mimicry in aposematic Dilophotes: Evidence for advergence and size-constraints in evolution of mimetic sexual dimorphism. Sci. Rep..

[37] Mallet J. (1995). A species definition for the modern synthesis. Trends Ecol. Evol..

[38] Dupuis J.R., Roe A.D., Sperling F.A.H. (2012). Multi-locus species delimitation in closely related animals and fungi: one marker is not enough. Mol. Ecol..

[39] Leache A., J Oaks J.R. (2017). The Utility of Single Nucleotide Polymorphism (SNP) Data in Phylogenetics. Ann. Rev. Ecol. Evol. Syst..

[40] Ivanov V., Lee K.M., Mutanen M. (2018). Mitonuclear discordance in wolf spiders: Genomic evidence for species integrity and introgression. Mol. Ecol..

[41] Tocco C., Dacke M., Byrne M. (2019). Eye and wing structure closely reflects the visual ecology of dung beetles. J. Comp. Physiol. A Neuroethol. Sens. Neur. Behav. Physiol..

[42] Mayr E. (1942). Systematics and the Origin of Species, from the Viewpoint of a Zoologist.

[43] Cracraft J. (1983). Species concepts and speciation analysis. Curr. Ornith..

[44] Coyne J.A., Orr H.A. (2004). Speciation.

[45] Mallet J. (2008). Hybridization, ecological races and the nature of species: Empirical evidence for the ease of speciation. Phil. Trans. R. Soc. Biol. Sci..

[46] Coates D.J., Byrne M., Moritz C. (2018). Genetic diversity and conservation units: dealing with the species-population continuum in the age of genomics. Front. Ecol. Evol..

[47] Rosser N., Freitas A.V.L., Huertas B., Joron M., Lamas G., Mérot C., Simpson F., Willmott K.R., Mallet J., Dasmahapatra K.K. (2019). Cryptic speciation associated with geographic and ecological divergence in two Amazonian Heliconius butterflies. Zool. J. Linn. Soc..

[48] Linck E., Epperly K., Van Els P., Spellman G.M., Bryson R.W., McCormack J.E., Canales-Del-Castillo R., Klicka J. (2019). Dense geographic and genomic sampling reveals paraphyly and a cryptic lineage in a classic sibling species complex. Syst. Biol..

[49] Elias M., Hill R.I., Willmott K.R., Dasmahapatra K.K., Brower A.V.Z., Mallet J., Jiggins C.D. (2007). Limited performance of DNA barcoding in a diverse community of tropical butterflies. Proc. R. Soc. Biol. Sci..

