# Supplementary material for: Genomic and Mitochondrial Data Identify Different Species Boundaries in Aposematically Polymorphic Eniclases Net-Winged Beetles (Coleoptera: Lycidae)
Source: Insects. 2019 Sep 11;10(9):295. doi: 10.3390/insects10090295 (PMC6780303; doi:10.3390/insects10090295)
Supplement: Supplementary file 1 [file insects-10-00295-s001.pdf]

Genomic and mitochondrial data identify different species boundaries in aposematically polymorphic *Eniclases* (Coleoptera: Lycidae)

Matej Bocek[1], Michal Motyka[1], Dominik Kusy[1], Ladislav Bocak[1]*

Supplements

Supplementary Text.
The brief taxonomic history, morphology, and diversity of *Eniclases* Waterhouse, 1879

Supplementary Tables

Table S1. The list of sampled localities with coordinates

Table S2. Euclidian distances among sampled localities in central New Guinea.

Table S3. The list of primers used for mtDNA amplification.

Table S4. Characteristics of datasets and best-fit models for mtDNA and nextRAD partitions.

Supplementary Figures

Maximum likelihood phylogenies of *Eniclases*. Nodes are colored according to ultrafast bootstrap values. The clades A, B, and C designate lineages of closely related species whose delimitation is discussed in the text.

Fig. S1. Topology recovered by the analysis of three mitochondrial DNA fragments, outgroups included, otherwise as Fig. 3A in the main text.

Fig. S2. The phylogenetic tree recovered by the analysis of *cox1* mtDNA fragment

Fig. S3. The phylogenetic tree recovered by the analysis of *rrnL* mtDNA fragment

Fig. S4. The phylogenetic tree recovered by the analysis of *nad5* mtDNA fragment

Fig. S5. Bayesian Poisson Tree Process species delimitation using cox1 mtDNA data (reprinted from Bocek & Bocak 2016)

Supplementary Text.

The brief taxonomic history, morphology, and diversity of *Eniclases* Waterhouse, 1879

The genus *Eniclases* was described by Waterhouse (1879) for a single species *Trichalus luteolus* Waterhouse, 1878 which was a year later described in *Lycus* (gen. 38). Further species were described by Kleine (1926, 1930, 1935). Additional two species were originally described in *Trichalus* by Pic (1921, 1923) and transferred to *Eniclases* by Bocak & Bocakova (1991). The later study additionally added a number of newly described species from New Guinea. The latest study dealing with *Eniclases* added further ten species, mainly from the central part of New Guinea (Bocek & Bocak 2016). and a new species was described from Halmahera (Bocek & Adamkova 2019).

Eniclases shares with other trichaline genera the shortened primary costa 1. Unlike related genera the pronotal carinae form a V-shaped pattern. The male genitalia are characterized by dorsal pigmented line of the phallus and are highly uniform in the whole genus. Therefore, the male genitalia could not be used for identification of species (Bocak & Bocakova, 1991). Most diagnostic characters have been found in the relative size of males eyes and the shape of male antennae. The detailed morphological characteristic of *Eniclases* was provided by Bocak & Bocakova (1991).

At present, *Eniclases* contains 37 species distributed mostly in New Guinea (35 spp.), two species is recorded from the Moluccas. Although Eniclases was earlier considered as a characteristic New Guinea lineage and the Moluccan record was supposed to be a result of a recent dispersal event, Bocek & Adamkova (2019) showed that the Moluccan species form a sister to all New Guinean species and that also Schizotrichalus, a supposed sister-lineage of *Eniclases*, occurs in the Moluccas. Eniclases were intensively collected only in two regions of New Guinea. American entomologists based in the Wau Ecological Institute assembled a large collection from the Owen's and Bismarck Ranges in 1950s and 1960s. The collection was studied in early 1990s (Bocak & Bocakova 1991). Further species were collected in the Central New Guinea in the Baliem valley and northern slopes of the Central Range and these were used for the current study. A few species were described from the Sepik area, Sentani/Jayapura (Hollandia), Manokwari (Dore Bay) and Raja Ampat islands (Misool).

The present analyses do not result in any formal taxonomical changes despite recovered alternative placement of some individuals (Fig. 4). The high intraspecific polymorphism and similarity makes morphology-based identification of *Eniclases* extremely difficult and in some cases neither morphology nor mitochondrial markers provide sufficient information for robust assignment of an individual to a species.

The list of species of *Eniclases* Waterhouse, 1879.

Eniclases Waterhouse, 1879: 66
Type species. *Trichalus luteolus* Waterhouse, 1878
Trichalus, subgenus *Trichalolus* Pic, 1923: 36; Bocak & Bocakova, 1991: 206
Type species. *Trichalus (Trichalolus) apertus* Pic, 1923: 36

apertus (Pic, 1923: 36)
Trichalus (Trichalolus) apertus Pic, 1923: 36
=*Eniclases fumosus* Kleine, 1926: 181; Bocak & Bocakova, 1991: 217
bicolor Bocek & Bocak, 2016: 23
bokondinensis Bocek & Bocak, 2016: 26
brancuccii Bocek & Bocak, 2016: 25
divaricatus (Pic, 1921: 10)
Trichalus divaricatus Pic, 1921: 10
efferatus Kleine, 1926: 181
egregius Kleine, 1926: 181
electus Kleine, 1926: 182
elelimensis Bocek & Bocak, 2016: 26
flabellatus Bocak & Bocakova, 1991: 207
flavoscutellaris Bocak & Bocakova, 1991: 216
fuscicornis Bocak & Bocakova, 1991: 208
infuscatus Bocek & Bocak, 2016: 23
luteolus (Waterhouse, 1878: 113)
Trichalus luteolus Waterhouse, 1878: 113
moluccanus Kleine, 1930: 328
nicricornis Bocak & Bocakova, 1991: 216
niger Bocek & Bocak, 2016: 29
nigriceps Bocak & Bocakova, 1991: 208
nigroruber Kleine, 1935: 318
pallidus Bocak & Bocakova, 1991: 209
papuensis Bocak & Bocakova, 1991: 213
pectinicornis Bocak & Bocakova, 1991: 211
pseudoapertus Bocek & Bocak, 2016: 21
pseudoluteolus Bocek & Bocak, 2016: 29
proximus Bocak & Bocakova, 1991: 209
riedeli Bocak & Bocakova, 1998: 14
robustus Bocak & Bocakova, 1991: 209
sedlaceki Bocak & Bocakova, 1991: 212
serratus Bocak & Bocakova, 1991: 217
similis Bocak & Bocakova, 1991: 210
slipinskii Bocak & Bocakova, 1991: 213
subelectus Bocak & Bocakova, 1991: 215
tikapurensis Bocek & Bocak, 2016: 24
variabilis Bocek & Bocak, 2016: 27
versicolor Kleine, 1926: 182
wauensis Bocak & Bocakova, 1991: 214

References

Bocak, L. Revision and phylogenetic analysis of Metriorrhynchinae. *European Journal of Entomology* **2002**, *99*, 315–351.

Bocak, L. A new species of the genus Eniclases Wat. (Coleoptera, Lycidae). *Acta Universitae Palackianae Olomucensis* **1998**, *35*(1997): 13–16.

Bocák, L.; Bocáková, M. Revision of the genus Eniclases Waterhouse, 1879 (Coleoptera, Lycidae, Metriorrhynchinae). *Mitteilungen der Münchner Entomologischen Gesellschaft* **1991**, *81*, 203–226.

Bocek, M.; Bocak, L. Where are species limits in polymorphic mimetic beetles from New Guinean mountains: a case of *Eniclases* net-winged beetles (Lycidae: Metriorrhynchini). *ZooKeys* **2016**, *593*, 15–35.

Bocek, M.; Adamkova, K. New species of trichaline net-winged beetles, with remarks on the phylogenetic position and distribution of *Schizotrichalus* (Coleoptera: Lycidae: Metriorrhynchinae). *Zootaxa*, **2019**, *4623*, 341–350.

Kleine, R. Coleoptera, Lycidae. *Nova Guinea*. *Résultats de l'expédition scientifique néerlandaise a la Nouvelle-Guinée* **1926**. *15*, 91–195.

Kleine, R. Bestimmungstabelle der Trichalusverwandschaft. *Treubia* 1930, 9, 325–340.

Kleine, R. Bericht uber die von Miss Chessman in British New Guinea gesammelten Brenthiden und Lyciden. *New Guinea*. *Résultats de l'expédition scientifique néerlandaise a la Nouvelle-Guinée* 1935 *17*. 303–322.

Pic, M. Contribution a l'étude des Lycides. *L'Echange* **1921**, *404*,1–4 (hors texte).

Pic, M. Contribution a l'étude des Lycides. *L'Echange* **1921**, *406*, 9–12 (hors texte).

Pic, M. Contribution a l'étude des Lycides. *L'Echange* **1923**, *412*, 36 (hors texte).

Waterhouse, C.O. On the different forms occurring in the Coleopterous family Lycidae, with descriptions of new genera and species. *Transactions of the Entomological Society London* **1878**: 95–118.

Waterhouse, C.O. *Illustration of the typical specimens of Coleoptera in the collection of the British Museum. Part I. - Lycidae*. British Museum, London. **1879**. 93 pp.

Table S1. The list sampled localities

West Papua Province, Manokwari regency			
Mokwam distr.			
Maibri vill., Arfak Mts.	1° 05'S	133° 54'E	1570 m
Papua Province			
Jayapura regency			
Sentani, Cyclop Mts.	02°32.320´S	140°30.738´E	360 m
Sentani, Cyclop Mts.	02°32.487´S	140°30.683´E	275 m
Tolikara regency			
Bokondini Distr.			
Bokondini	3°40.76'S	138°40.15'E	1287 m
Bokondini	3°40.76´S	138°40.15´E	1250–1300 m
3 km N Bokondini	3°39.741´S	138°40.216´E	1750–1900 m
3 km SW Bokondini	3°42.51´S	138°38.893´E	2100 m
Yalimo regency			
Elelim, km 5 rd Apalapsili	3°48.700´S	139°22.088´E	580 m
Elelim, km 6 rd Apalapsili	3°48.686´S	139°21.764´E	650 m
Dombomi, Lower Pass vall.	3°49.477´S	139°10.251´E	1150 m
Central Mamberamo Regency			
Kelila distr.			
Tikapura (Rd Tagime-Kelila)	3°46.797'S	138°42.933´E	2170 m
Jayawijaya Regency			
Wamena distr.			
Yiwika, 16 km N Wamena	3°56.883´S	138°57.712´E	2100 m

Table S2. Euclidian distances between *Eniclases* localities in kilometers. Aggregations designated as AGG1–4, see main text for further information.

	Sentani 275m	Elelim 580m	Domb 1150m	Bok1 1287m	Bok2 1250m	BokN 2100m	BokSW 1900m	Tikap 2150m	Yiwika 2100m
Sentani	-								
Elelim	189.3	-							
Dombomi	205.8	22.1	-						
Bokondini1	240.3	79.1	58.0	-					
Bokondini2	240.4	79.4	58.3	0.3	-				
Bokondini N	239.3	79.4	58.5	1.9	1.8	-			
Bokondini SW	244.0	80.9	59.5	4.0	4.0	5.7	-		
Tikapura	242.1	72.7	50.8	12.3	12.5	14.0	10.7	-	
Yiwika	232.1	47.7	27.0	44.2	44.3	45.2	43.8	33.8	-

Table S3. The list of primers used for mtDNA amplification.

Fragment Code	-mer	Sequence (5’ >> 3’)
rrnL		
16a	20	CGCCTGTTTAACAAAAACAT
ND1A	27	GGTCCCTTACGAATTTGAATATATCCT
ND1-2	24	ATCAAAAGGAGCTCGATTAGTTTC
cox1		
JerryN	23	CAACAYYTATTYTGATTYTTYGG
MarcyN	24	TTCRTAWGTTCARTATCATTGRTG
JerryM	23	CAACAYYTATTTTGRTTYTTTGG
Marcy	27	TARTTCRTATGWRCAATAYCAYTGRTG
SPat	21	GCACTAWTCTGCCATATTAGA
SJerry	23	CAACATYTATTYTGATTYTTTGG
Pat	25	TCCATTGCACTAATCTGCCATATTA
Jerry	23	CAACATTTATTTTGATTTTTT
Marilyn	21	TCATAAGTTCAGTATCATTG
nad5		
OF1	29	CCTACTCCTGTTTCTGCTTTAGTTCATTC
R6	29	GAAACGAAAAATCGTATTTAATTTCGACT
R2M	29	AATTGAASCCAAAAAGAGGTATATCACTG

Table S4. Characteristics of datasets and best-fit models for mtDNA and nextRAD partitions.

mtDNA dataset analysis

Fragment Name	Number of sequences	Sites	Unique	Informative	Constant
rrnL	63	796	99	83	679
cox1	64	1101	227	249	807
nad5	62	1207	272	299	864

Name	Model	LogL	AIC	w-AIC	AICc	w-AICc	BIC	w-BIC
rrnL	TIM3+F+I	-1983.31	4226.64	+ 0.0000	4277.86	+ 0.0000	4834.9869	+ 0.0000
cox1	TIM2+F+I+G4	-4175.6	8617.17	+ 0.0000	8654.03	+ 0.0000	9282.6984	+ 0.0000
nad5	HKY+F+I+G4	-4303.48	8860.96	+ 0.0000	8891.09	+ 0.0000	9508.1350	+ 0.0000

nextRAD dataset alignment analysis (Wclust = 0.85, MinCov = 4).

Input data: 66 sequences with 6066010 nucleotide sites
Number of constant sites: 5.58659 x 10^6 (= 92.0966 % of all sites)
Number of invariant (constant or ambiguous constant) sites: 5.58659 x 10^6 (= 92.0966 % of all sites)
Number of parsimony informative sites: 154300
Number of distinct site patterns: 942024

Model of substitution: GTR+F+I+G4

Rate parameter R:
A-C: 0.9833
A-G: 3.6524
A-T: 0.9157
C-G: 0.9335
C-T: 3.7609
G-T: 1.0000

	State frequencies	Rate matrix Q:			
A	pi(A) = 0.2936	-0.9256	0.1451	0.6	0.1806
C	pi(C) = 0.2047	0.208	-1.103	0.1533	0.7419
G	pi(G) = 0.2279	0.7727	0.1377	-1.108	0.1973
T	pi(T) = 0.2737	0.1937	0.5548	0.1643	-0.9128

Model of rate heterogeneity: Invar+Gamma with 4 categories
Proportion of invariable sites: 0.6185
Gamma shape alpha: 0.7601

Category	Relative_rate	Proportion
0	0	0.6185
1	0.2282	0.09538
2	1.024	0.09538
3	2.478	0.09538
4	6.755	0.09538

Relative rates are computed as MEAN of the portion of the Gamma distribution falling in the category.

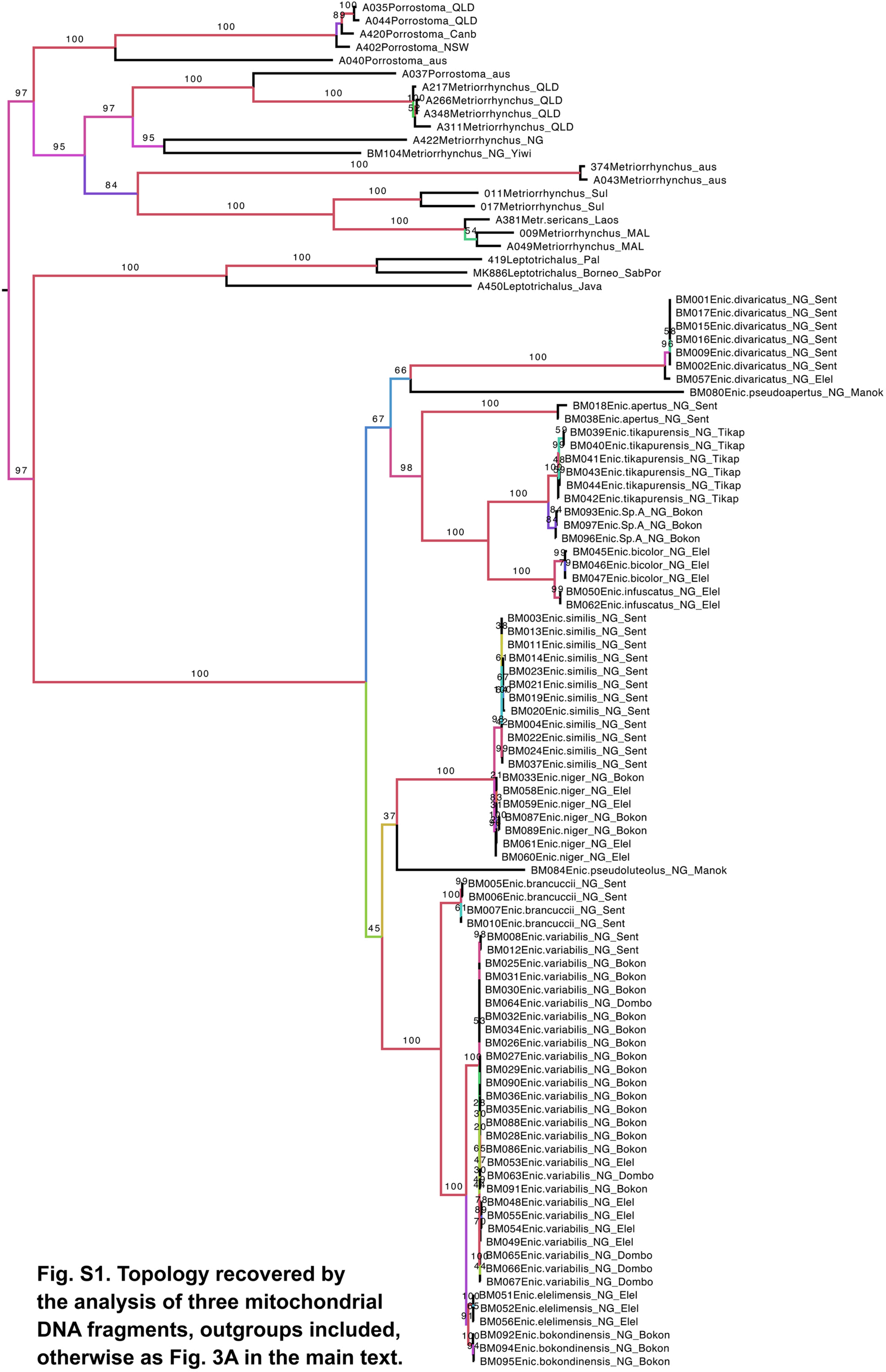

Fig. S1. Topology recovered by the analysis of three mitochondrial DNA fragments, outgroups included, otherwise as Fig. 3A in the main text.

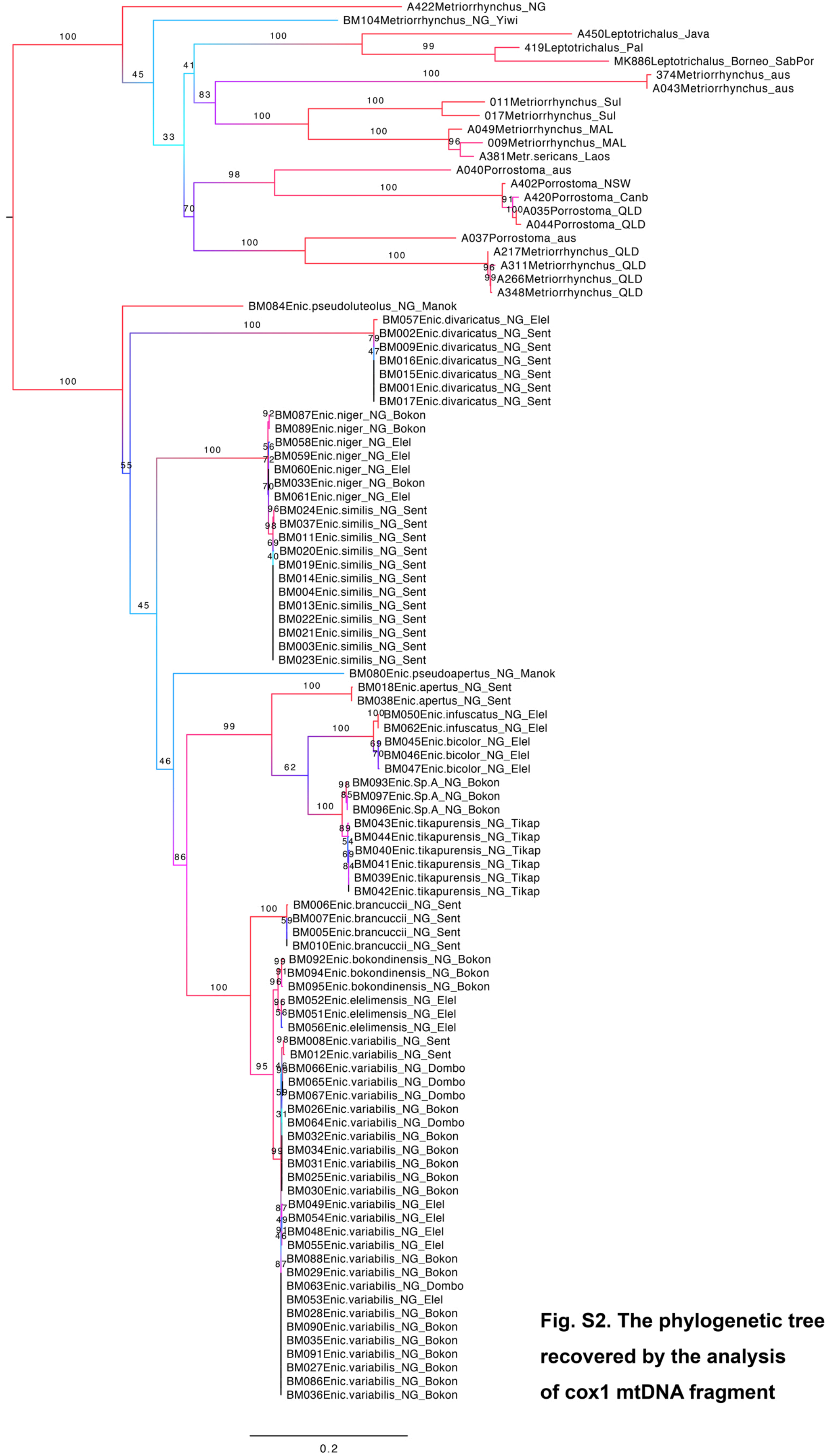

Fig. S2. The phylogenetic tree recovered by the analysis of cox1 mtDNA fragment

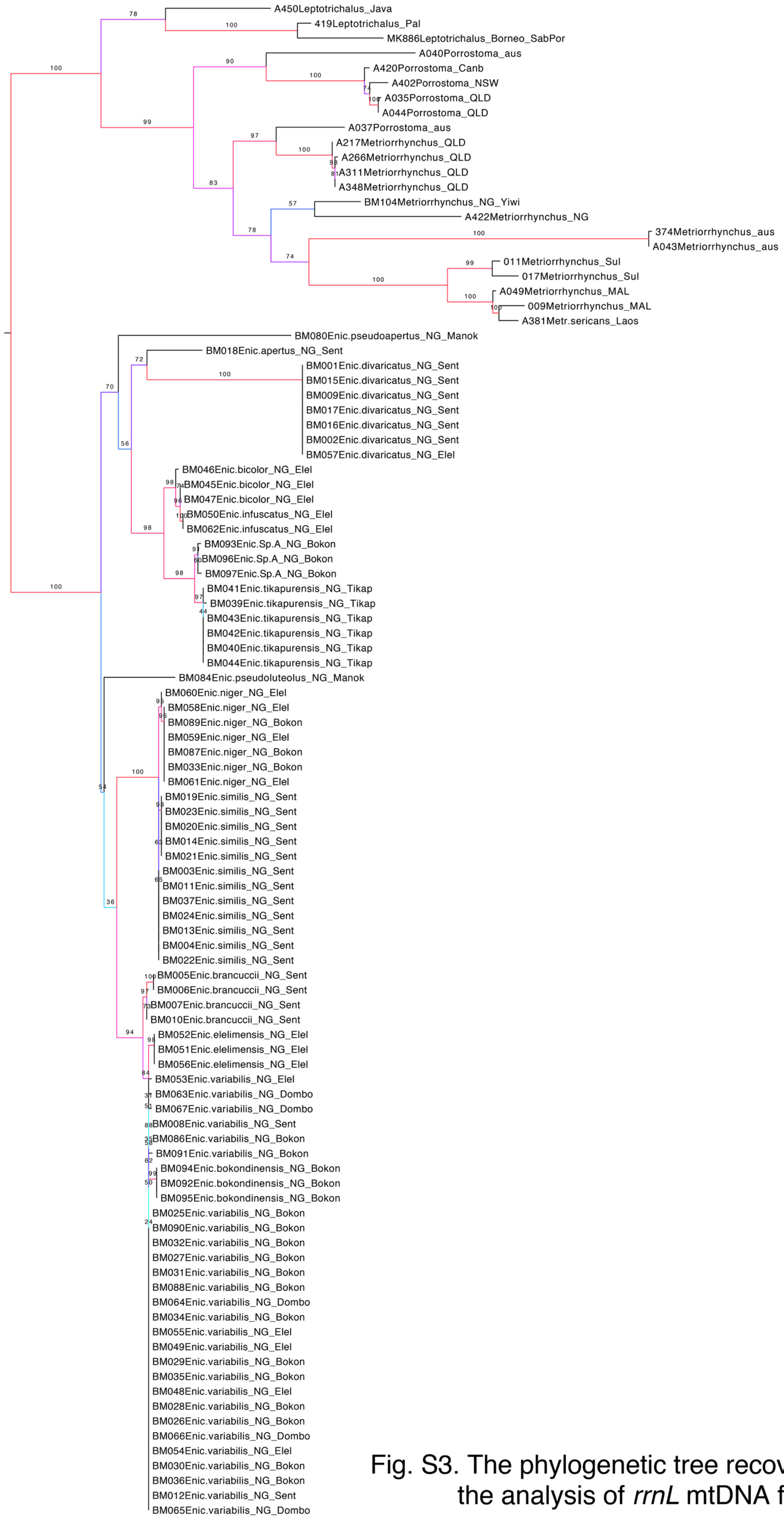

Fig. S3. The phylogenetic tree recovered by the analysis of *rrnL* mtDNA fragment

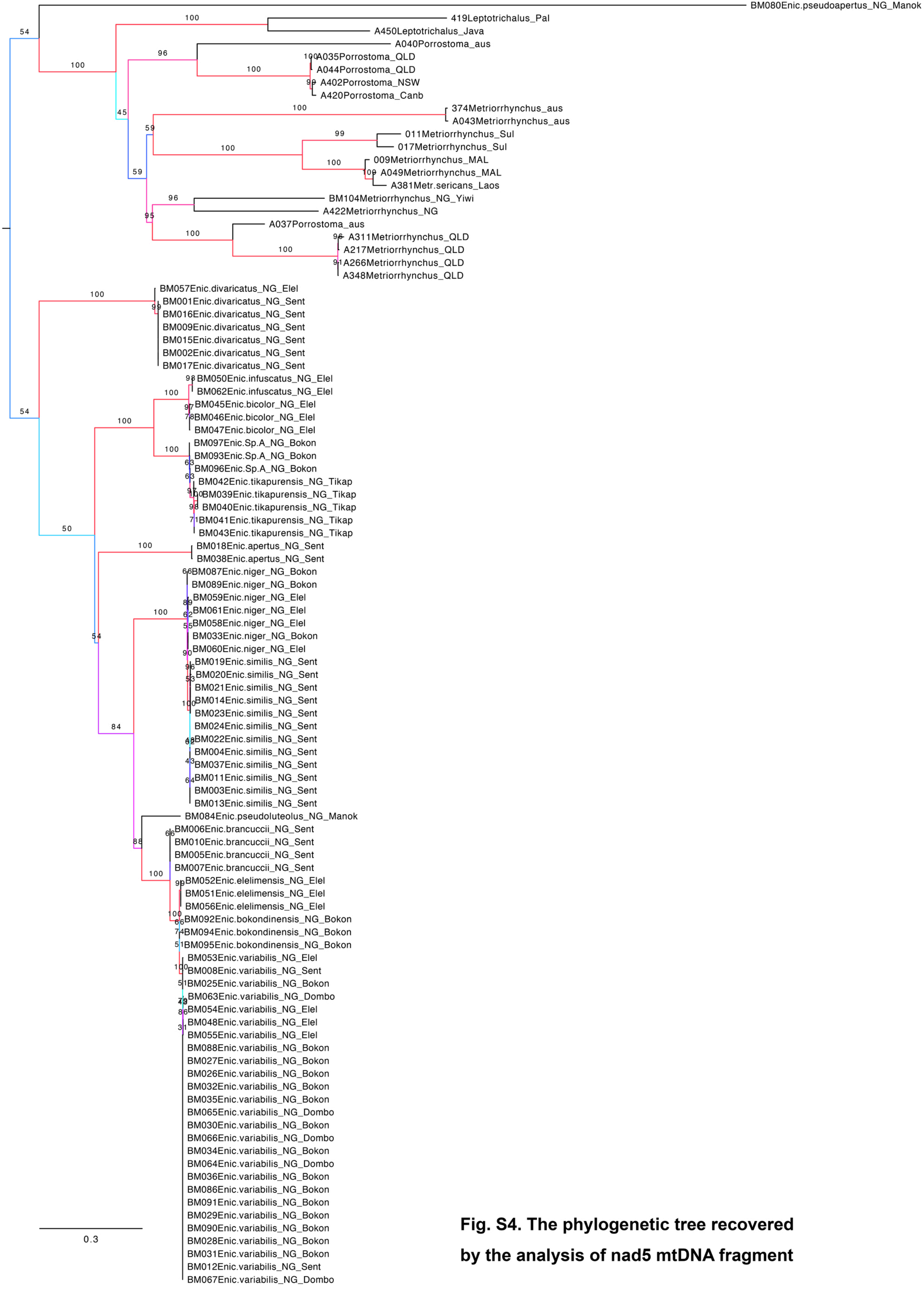

Fig. S4. The phylogenetic tree recovered by the analysis of nad5 mtDNA fragment

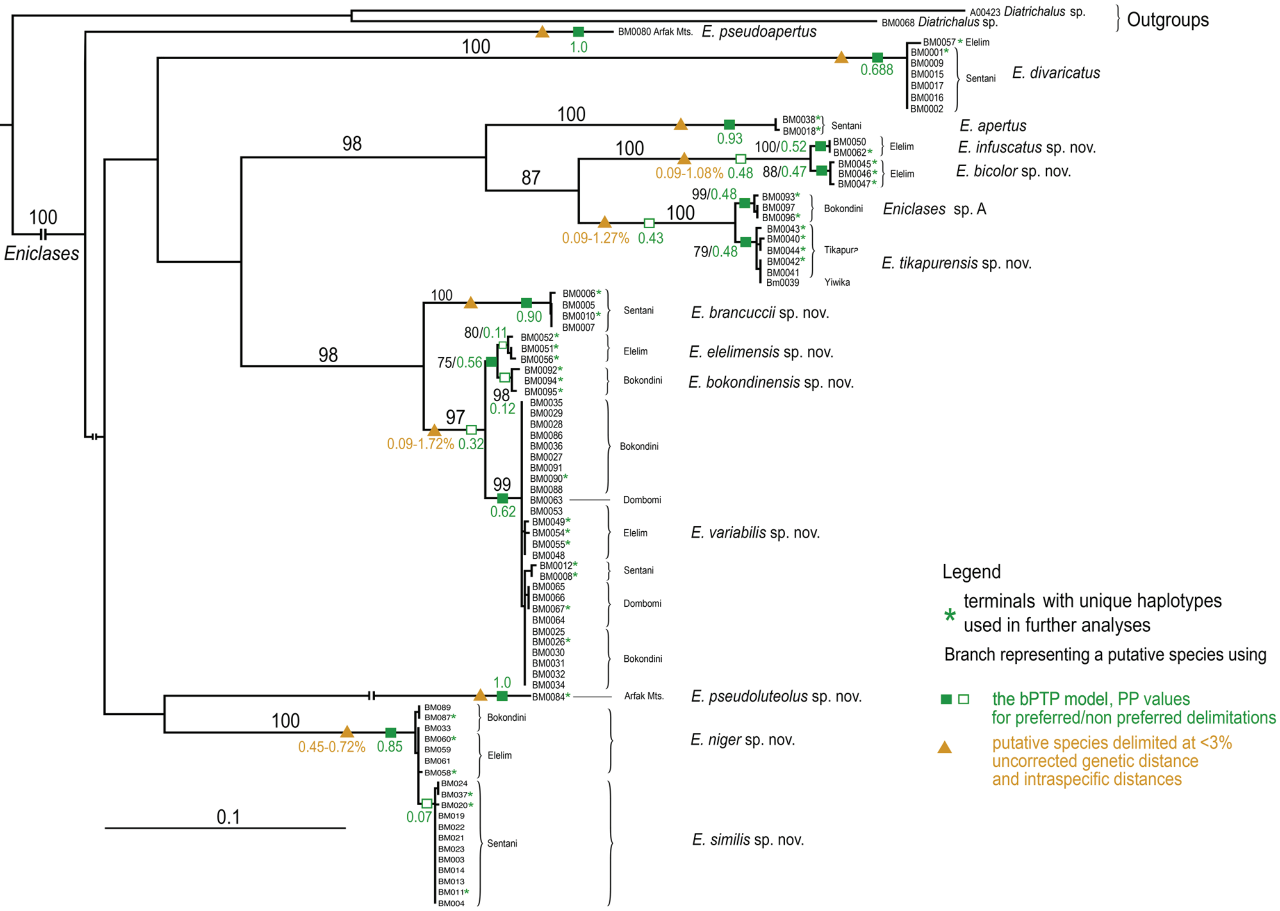

Figure S5. Bayesian Poisson Tree Process species delimitation using *cox1* mtDNA data (reprinted from Bocek & Bocak 2016)
